# Optimized Feature Extraction and Multi-Scale Fusion for Lightweight RTDETR in Real-Time Morphological Quality Detection of Oyster Mushroom (*Pleurotus ostreatus*) Toward Edge Deployment

**DOI:** 10.3390/foods15142429

**Published:** 2026-07-08

**Authors:** Zhuo Bai, Xuexi Qi, Yinyi Zhang, Yindi Xu, Chengnan Ru, Shuai Wang, Ziyue Li, Qiyuan Fu, Lei Shi, Yuxin Ye

**Affiliations:** 1 Institute of Smart Agriculture, Jilin Agricultural University, Changchun 130118, China; 2 College of Information Technology, Jilin Agricultural University, Changchun 130118, China; 3 College of Computer Science and Technology, Jilin University, Changchun 130012, China

**Keywords:** *Pleurotus ostreatus*, object detection, RTDETR, channel pruning, edge deployment

## Abstract

To address the low efficiency of manual quality grading for *Pleurotus ostreatus* in factory-scale production and the difficulty existing computer vision models face in balancing high localization accuracy with real-time edge deployment for food processing, a lightweight non-destructive detection model named POC-DETR-Prune is proposed. Based on an improved RTDETR framework, FasterNet is introduced to optimize feature extraction, reducing memory access latency while ensuring deep feature representation for complex food morphologies. A Small Object Enhancement Pyramid (SOEP) module is designed to mitigate the loss of subtle features caused by dense mushroom clustering. Furthermore, the Inner-MPDIoU loss function is proposed to significantly improve bounding box localization accuracy in highly overlapped food sorting scenarios. To adapt to industrial hardware constraints, a Random channel pruning strategy compresses computational overhead. Experimental results demonstrate that POC-DETR-Prune achieves a mAP@0.5:0.95 of 83.7% with a computation load of only 38.2 GFLOPs. Deployment testing on the NVIDIA Jetson Orin Nano Super edge computing platform achieves a real-time detection rate of 30.2 FPS. This emerging technology provides a certain level of visual algorithm support for automated quality grading equipment in the edible fungi industry.

## 1. Introduction

*Pleurotus ostreatus* is a highly nutritious edible fungus with increasing global consumption, making its automated processing a priority for the modern food industry [1,2]. With the transition towards Food and Agriculture 4.0, integrating emerging technologies like artificial intelligence into post-harvest processing is crucial for building efficient eco-agricultural systems. However, the real-time quality grading of *Pleurotus ostreatus* in factory environments relies heavily on manual labor, which is inefficient and highly subjective. While machine vision has been applied to edible fungi—such as using YOLO models for detecting shiitake and enoki mushrooms—real-time morphological grading faces critical scientific challenges. Densely clustered, highly overlapped, and extremely irregularly shaped fruiting bodies against complex backgrounds are difficult to process at high speeds. Existing object detection models struggle to balance subtle defect capture with the computational efficiency required for edge devices on food sorting lines. To overcome these bottlenecks, this paper proposes POC-DETR-Prune, a lightweight, non-destructive detection technology based on an improved Real-Time Detection Transformer (RTDETR), aiming to achieve high-precision, automated morphological quality grading for the food manufacturing sector.

With the advancement of smart agriculture, several scholars have currently conducted research on machine vision processing related to edible fungi. Qi et al. [3] performed detection and classification of shiitake mushroom fruiting bodies based on Mamba-YOLO; the results showed that the classification accuracies for categories including mushroom stick, flat immature, flat mature, cracked surface immature, cracked surface mature, deformed mature, and deformed immature shiitake were 98.1%, 98.3%, 98.2%, 98.8%, 98.5%, 96.2%, and 96.9%, respectively, thereby conducting meticulous information processing and research on various conditions of shiitake fruiting bodies; Shi et al. [4] utilized multiple lightweight methods to improve YOLOv8n, constructing OMB-YOLO-tiny for detecting four types of damage in *Pleurotus ostreatus*, ultimately surpassing mainstream models in both accuracy and inference speed while reducing parameters by nearly half, thereby conducting various lightweight attempts and combinatorial innovations in the edible fungi domain; Xie et al. [5] proposed an improved method that enhanced YOLOv8n-seg through StarNet, SPPECAN, and C2f DStar, and introduced a mask ownership judgment and merging optimization algorithm to correct position offset, internal disconnection, and boundary instability in grasping region prediction, thus addressing the deformation problem in enoki mushroom due to the inability to adjust grasping force based on individual mushroom size; the final mean average precision (mAP50:95) for grasping region segmentation was 0.743, representing a 4.5% improvement over YOLOv8, with an average detection speed of 10.3 milliseconds and a target width measurement error of only 0.14%. The proposed mapping relationship achieved adaptive end-effector control, thus realizing a 96% grasping success rate and a 98% qualified cutting surface rate, integrating machine vision with traditional mechanics. Shi et al. [6] proposed a lightweight model, OMC-YOLO, based on the improvement of YOLOv8n for the automated detection and grading of *Pleurotus ostreatus*; targeting the problems of low efficiency, high cost, and difficult manual quality assurance in traditional cultivation, they refined OMC-YOLO based on the YOLOv8n model, ultimately achieving a mAP@0.5 of 94.95%, an improvement of 2.62%, while reducing parameter count and computational load by 26%, thus providing technical support for automatic oyster mushroom grading, facilitating quality control and reducing labor costs, and holding positive significance for smart agriculture development; however, this work was limited to the grading of individual mushroom pieces and did not conduct a holistic morphological quality grading of the entire cluster. Although numerous scholars have achieved certain results in machine vision processing related to edible fungi, the real-time quality grading of *Pleurotus ostreatus* in factory cultivation environments still faces a key scientific challenge: how to accurately parse the morphological characteristics of densely clustered, highly overlapped, and extremely irregularly shaped fruiting bodies against complex backgrounds while maintaining extremely high detection speed. Existing object detection models [7] often struggle to strike a balance between small target capture capability and computational resource consumption when handling such tasks, and traditional bounding box regression loss functions are prone to localization accuracy bottlenecks when confronting the blurred edge textures and irregular overlapping of oyster mushrooms. Furthermore, how to deploy high-precision deep Transformer models on resource-constrained embedded edge devices without significantly sacrificing their perceptual capability remains an urgent engineering science problem to be solved in the intelligent production of edible fungi [8].

To address the aforementioned issues, this paper proposes a lightweight model, POC-DETR-Prune, based on an improved Real-Time Detection Transformer (RTDETR), aimed at achieving high-precision real-time grading of the morphological quality of *Pleurotus ostreatus*. To explicitly align the detection output with commercial grading decisions in actual production, this model utilizes bounding box object detection to evaluate and score the overall morphological quality of the entire fruiting body cluster (as a complete production and harvesting unit), rather than grading isolated individual mushrooms. The main contributions of this study are as follows:

1. An efficient feature extraction network based on FasterNet was constructed: by introducing Partial Convolution (PConv) to optimize memory access efficiency, the model’s inference latency is significantly reduced while ensuring the strength of deep semantic feature extraction.
2. A Small Object Enhancement Pyramid (SOEP) module was designed: utilizing SPDConv to losslessly mAP high-resolution features from the P2 layer, combined with the OmniKernel multi-branch attention mechanism, effectively addressing the loss of small target features caused by the early growth stage and dense clustering and occlusion of *Pleurotus ostreatus* [9].
3. The Inner-MPDIoU loss function was proposed: innovatively integrating the scale-adaptive auxiliary bounding box strategy with the minimum point distance optimization method, significantly improving the model’s convergence speed and localization accuracy when dealing with highly overlapped grading scenarios.
4. A Random channel pruning strategy based on the “Lottery Ticket Hypothesis” was implemented: on the premise of maintaining detection accuracy, the computational load was successfully compressed to 38.2 GFLOPs, demonstrating the effectiveness and advancement of this lightweight strategy in agricultural edge computing scenarios with limited computational resources.

## 2. Materials and Methods

### 2.1. Establishment of the Image Dataset

The original image dataset comprised 1911 images of *Pleurotus ostreatus*, collected from six independent cultivation batches. Images were captured using high-resolution smartphone cameras (a 12-megapixel iPhone 13 and a 50-megapixel Xiaomi 14) under natural cultivation conditions to approximate the imaging conditions of automated sorting equipment. During the training phase, all images were uniformly resized to 640 × 640 pixels.

**Figure 1:**
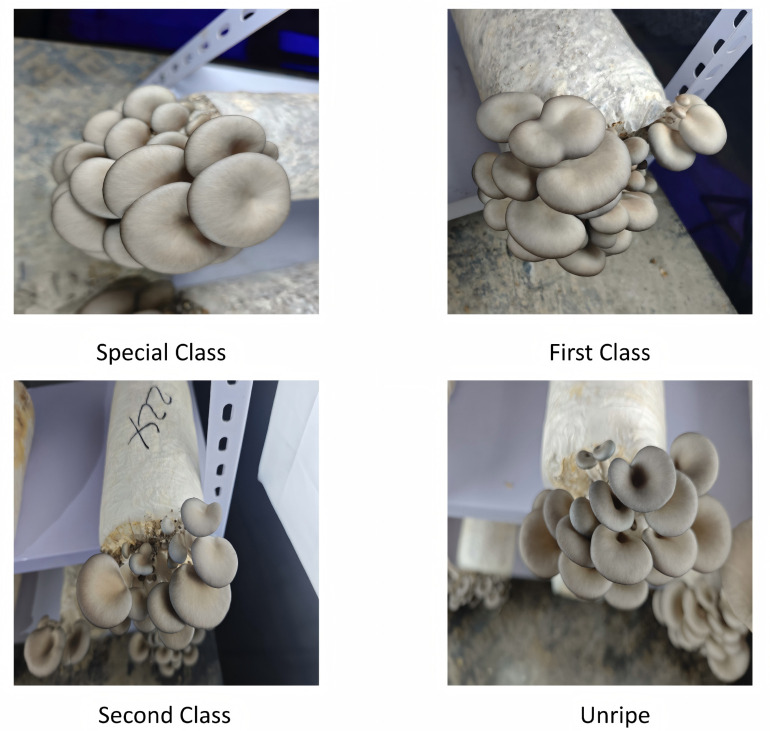
Schematic diagrams of Special Class, First Class, Second Class, and Unripe of *Pleurotus ostreatus*.

**Figure 2:**
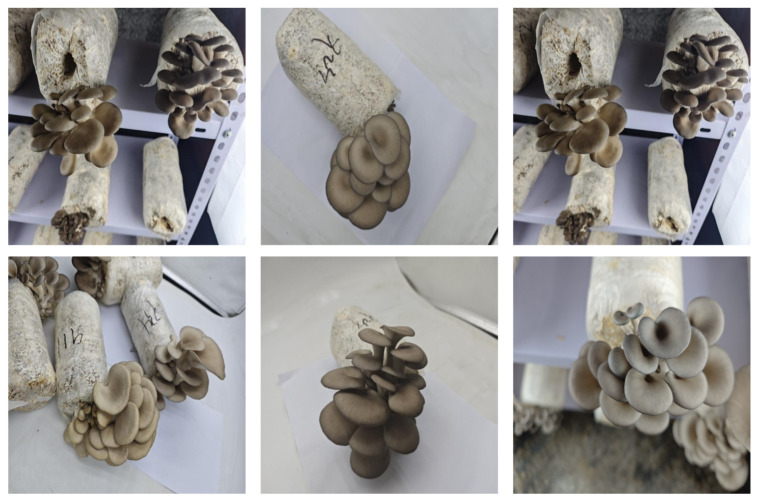
Representative images from the dataset illustrating variations in illumination, viewpoints, cultivation layouts, and background environments.

The collected images were manually annotated using the MakeSense tool according to the national quality grading standards for *Pleurotus ostreatus* of the People’s Republic of China (Table 1). Figure 1 presents representative examples of the four quality categories (Special Class, First Class, Second Class, and Unripe fruiting bodies), illustrating the morphological characteristics used for quality grading. Figure 2 further presents representative images from the dataset acquired under different illumination conditions, camera viewpoints, cultivation layouts, and background environments. Variations in target density, occlusion level, and fruiting body size can also be observed, providing an overview of the variability present in the collected dataset.

To ensure annotation consistency and minimize subjective bias, one independent annotator labeled the dataset, and a second expert reviewed and resolved disagreements in both category assignment and bounding box annotation.

**Figure 3:**
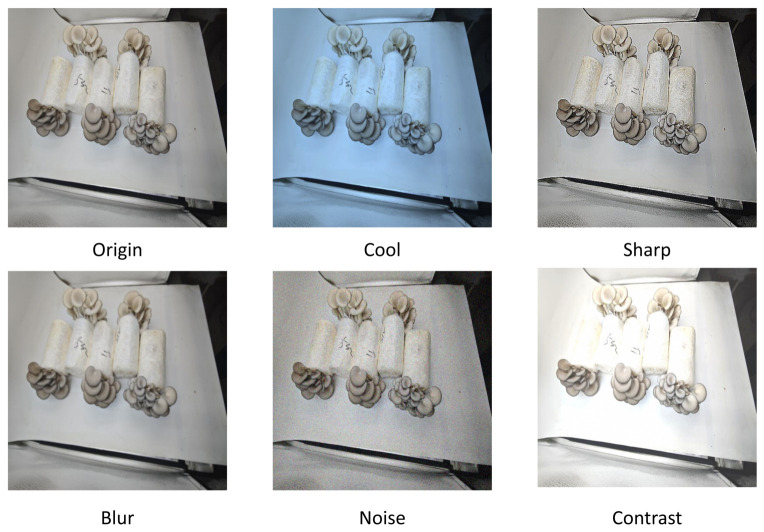
Display of data augmentation effects.

Image preprocessing is crucial for enhancing data diversity and improving model training efficacy. To rigorously prevent data leakage and avoid artificially inflated performance metrics, the original dataset was first partitioned into training, validation, and test sets in an 8:1:1 ratio prior to any image manipulation [10]. Data augmentation techniques—including brightness adjustment, blurring, and sharpening (see Figure 3)—were exclusively applied to the training set. The validation and test sets remained strictly composed of unaugmented, original images. Annotation consistency was maintained by synchronizing the corresponding label files during the augmentation process [11].

The original dataset exhibited an imbalanced class distribution. Following the targeted augmentation strategy on the training subset, the final comprehensive dataset reached a total of 5040 images. This effectively balanced the categories, laying a solid foundation for model training and evaluation while preventing issues such as overfitting [12]. The detailed distribution of the dataset before and after augmentation is presented in Table 2.

### 2.2. RTDETR and POC-DETR-Prune

The Real-Time Detection Transformer (RTDETR) represents a significant advancement in the field of computer vision, effectively applying the Transformer architecture to real-time object detection and addressing the long-standing trade-off between speed and accuracy that exists between traditional Convolutional Neural Network (CNN)-based detectors and Transformer-based detectors [13,14,15]. Prior to the introduction of RTDETR, the real-time detection domain had long been dominated by CNN-based architectures such as the YOLO series; although these models exhibited excellent inference speed, their performance was often constrained by the post-processing step of Non-Maximum Suppression (NMS), which not only introduced additional inference latency and hyperparameter instability but also hindered the achievement of true end-to-end optimization. In contrast, although the DETR series models achieved end-to-end detection by introducing object queries and bipartite graph matching, thereby eliminating the dependency on NMS, the high computational cost incurred when processing high-resolution multi-scale features severely constrained their inference speed, making them difficult to apply in latency-critical scenarios such as autonomous driving or industrial real-time quality inspection. RTDETR broke this impasse through a series of ingenious architectural innovations, with its core contribution being the proposal of an Efficient Hybrid Encoder, which creatively decouples intra-scale feature interaction and cross-scale feature fusion; it employs an Attention-based Intra-scale Feature Interaction (AIFI) module to perform global modeling exclusively on high-level feature layers to capture deep semantic information, while simultaneously utilizing a CNN-based Cross-scale Feature Fusion (CCFF) module to efficiently aggregate multi-scale contexts, thereby substantially reducing computational complexity while retaining the advantage of Transformers in processing global information. Furthermore, addressing the issues of inaccurate query initialization and slow convergence prevalent in DETR-like models, RTDETR introduced an IoU-aware Query Selection mechanism, which, through soft label constraints during the training phase, explicitly utilizes IoU scores to guide the selection of encoder features, thereby ensuring that features initialized as object queries can align more accurately with target regions, significantly improving localization precision and model convergence efficiency. It is worth mentioning that RTDETR also possesses flexible inference speed adjustability, allowing it to adapt to different hardware computational constraints by dynamically adjusting the number of decoder layers without the need for retraining, demonstrating substantial practical engineering value. Extensive experiments on authoritative benchmark datasets such as COCO have demonstrated that RTDETR, while maintaining real-time frame rates, demonstrates competitive detection accuracy (AP) compared to state-of-the-art (SOTA) models of equivalent scale such as YOLOv8. This performance highlights the potential of pure Transformer architectures in the real-time detection domain and provides a valuable reference for future research on end-to-end universal visual perception systems.

**Figure 4:**
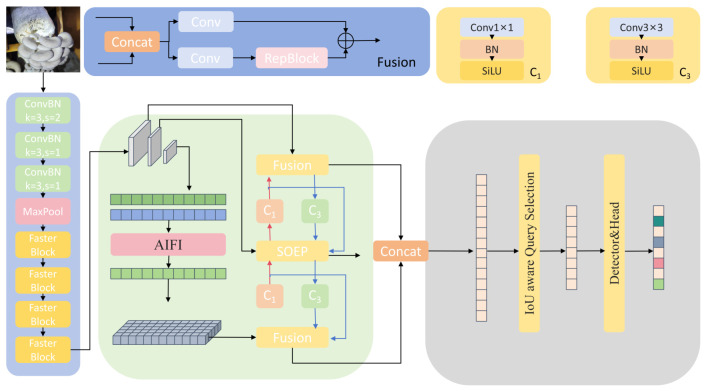
Network architecture diagram of POC-DETR-Prune.

POC-DETR-Prune is a high-precision lightweight detection model deeply customized and optimized on the basis of the RTDETR architecture, specifically targeting the challenges present in the morphological quality detection of *Pleurotus ostreatus* fruiting bodies, such as dense occlusion, the ease of losing small target features, and the high real-time requirements for industrial deployment. Although RTDETR addressed the real-time issue through the hybrid encoder, when confronting agricultural targets like *Pleurotus ostreatus* with highly variable morphology and complex growth environments, its original backbone network still exhibited deficiencies in feature extraction efficiency and small target capture capability, while its high computational overhead restricted its application on edge devices. To this end, POC-DETR-Prune first introduced FasterNet as the feature extraction core of the backbone network; this module innovatively adopts Partial Convolution (PConv) technology, which effectively reduces redundant computation and frequent memory accesses by decoupling spatial and channel correlations, thereby solving the problem in traditional CNNs where low FLOPs cannot be directly translated into high inference speed (FLOPS), providing the model with more efficient and low-latency low-level feature representation capabilities. Targeting the issues of minute fruiting bodies in the early growth stage and feature blurring caused by multiple clusters growing together, the model constructed a Small Object Enhancement Pyramid (SOEP) module; instead of adopting the traditional crude downsampling approach, this module utilizes SPDConv to losslessly mAP the high-resolution features of the P2 layer into deep networks, combined with the OmniKernel multi-branch attention mechanism (including global, large kernel, and local branches), achieving precise capture and multi-scale fusion of semantic information and spatial details of small targets, effectively resolving the deficiency of RTDETR in losing small object information in deep feature maps. At the level of model training optimization, given that the edges of *Pleurotus ostreatus* fruiting bodies are irregular and severely overlap with each other, making it difficult for traditional IoU loss functions to achieve refined bounding box regression, POC-DETR-Prune proposed the Inner-MPDIoU loss function, which creatively integrates the scale-adaptive auxiliary bounding box strategy of Inner-IoU with the minimum point distance optimization method of MPDIoU, not only accelerating model convergence using auxiliary bounding boxes but also significantly enhancing localization accuracy and robustness in high-overlap scenarios through point distance constraints. Finally, to further adapt to the hardware constraints of agricultural fields, the model adopted a Random channel pruning strategy; by randomly eliminating redundant channels combined with fine-tuning training, the model’s computational complexity was successfully reduced to 38.2 GFLOPs, maintaining a high average precision of 83.7% (mAP@0.5:0.95) while significantly compressing parameter count, thus achieving a favorable balance among detection accuracy, inference speed, and model lightweighting, providing an effective and practical technical solution for real-time quality monitoring in the intelligent production of edible fungi.POC-DETR-Prune network architecture diagram (see Figure 4).

### 2.3. FasterNet

FasterNet is a neural network architecture proposed by the Hong Kong University of Science and Technology, designed to pursue faster neural network training and inference speeds. Addressing the question of where the faster speed of neural networks is manifested, it can be understood from the following formula that network latency is determined by two factors; consequently, to accelerate the computational speed of a neural network, one can either increase the value of FLOPS or decrease the value of FLOPs.

(1) $$Latency=\frac{FLOPs}{FLOPS}$$

Here, FLOPS (Floating-point Operations Per Second) refers to the number of floating-point operations that can be executed per second; FLOPs (Floating-point Operations) refers to the total number of floating-point operations required by a task, which can be understood as the computational workload of a task; and Latency refers to the delay in network computation, i.e., the time required for the network to process a task, which can be understood as the computational speed of the neural network. Latency is determined by the ratio of FLOPs to FLOPS.

**Figure 5:**
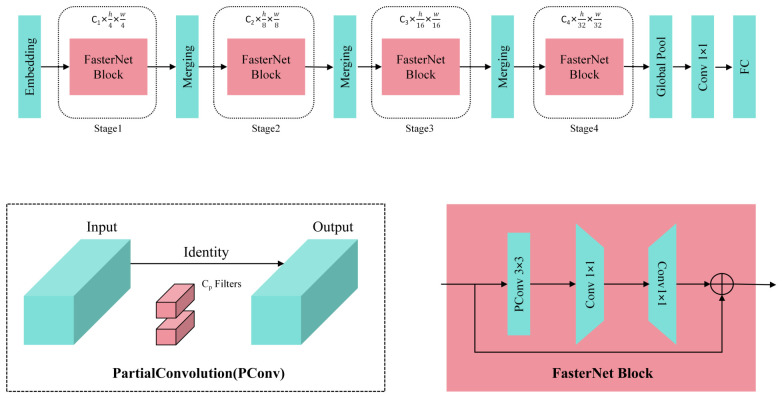
Network architecture of FasterNet.

Through research on the operations of neural network operators, Jierun Chen et al. [16] discovered that the primary cause of low network computation speed is the excessively frequent access of operators to hardware memory during network computation; frequent access to hardware memory reduces the efficiency of the hardware in processing computational tasks, thereby decreasing the value of FLOPS. To reduce the number of memory accesses during computation and achieve the goal of reducing latency by increasing the FLOPS value, FasterNet proposed the concept of Partial Convolution (PConv); by adopting partial convolution and an inverted residual structure, the number of accesses to hardware memory during computation was reduced, lowering the memory access cost of the network and improving its computational efficiency. Figure 5 illustrates the network architecture of FasterNet.

The feature extraction network of FasterNet is roughly divided into four parts, each consisting of an embedding layer or a merging layer plus a FasterNetBlock. The embedding layer employs a convolutional layer with a stride of 4 and a kernel size of 4 × 4 for spatial downsampling, serving the function of reducing the network’s computational parameter count; the merging layer uses a convolutional layer with a stride of 2 and a kernel size of 2 × 2, primarily functioning to expand the number of channels. The FasterNetBlock employs Partial Convolution (PConv) and regular 1 × 1 convolutions to form an inverted residual structure; the first convolutional layer uses PConv, followed by two 1 × 1 convolutions, and the input image information is added to the initial input data after passing through the final 1 × 1 convolution to produce the network output.

### 2.4. SOEP

**Figure 6:**
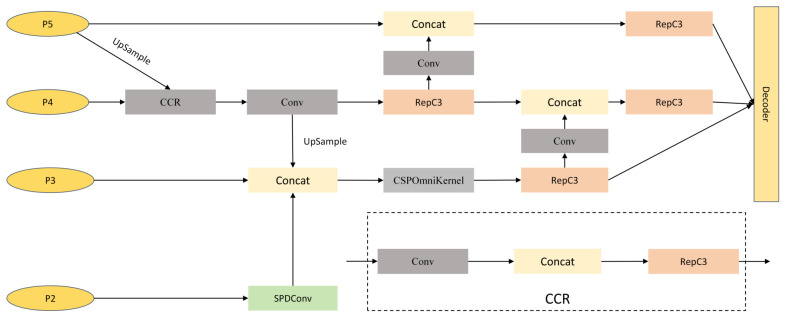
Structure of the SOEP module.

The small targets captured in the daily photography of *Pleurotus ostreatus* in this study appear somewhat challenging on the conventional P3, P4, and P5 detection layers; one of the reasons is that some small target samples in our dataset are relatively small in size, while the downsampling factor in RTDETR is relatively large, resulting in feature loss for small targets, and making it difficult for deeper feature maps to learn the feature information of small targets. A currently widespread approach is to add a P2 detection layer to enhance the detection capability for small targets. The P2 detection layer effectively captures subtle features through deeper feature processing, thereby improving the detection accuracy for small targets. Furthermore, the efficient feature fusion mechanism introduced by the P2 detection layer can combine low-level features (such as rich positional information) with high-level features (such as abstract semantic information), further enhancing the localization and classification capabilities for small targets. This improvement significantly enhances the detection performance of RTDETR for small targets in complex scenes. However, it simultaneously introduces a series of problems; for instance, adding the P2 detection layer leads to issues such as excessive computational load and more time-consuming post-processing, which increasingly motivates researchers to develop new effective feature pyramids specifically for small targets. This paper proposes SOEP to enhance the detection capability for small targets. The following describes the specific implementation process of SOEP in RTDETR, with the module structure of SOEP illustrated in Figure 6.

**Figure 7:**
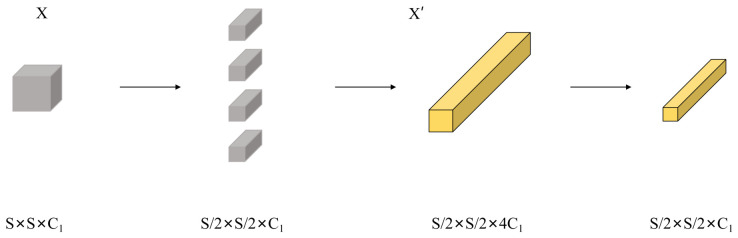
Structure diagram of SPDConv.

First, high-resolution feature maps (160 × 160) from the P2 layer are extracted from the backbone network; these feature maps retain rich details and positional information of small targets. To ensure that this detailed information is not lost in the subsequent detection process, the P2 feature layer undergoes SPDConv processing to transmit features rich in small target information to the P3 layer for fusion. SPDConv extracts key features of small targets through convolution operations and performs scale adjustment, enabling these features to effectively fuse with the P3 layer features. The working principle of SPD-Conv can be summarized in the following two steps: first, a space-to-depth transformation is performed, converting the spatial dimensions of the feature map into the depth dimension. Specifically, for a feature map *X* of size $S\times S\times C_{1}$, where *S* is the length and width, and $C_{1}$ is the number of channels, four sub-mAPs are obtained through slicing operations, each of size $S/2\times S/2\times C_{1}$. These four sub-mAPs are concatenated along the channel dimension to obtain a feature map $X^{\prime }$ of size $S/2\times S/2\times 4C_{1}$. Subsequently, a non-strided convolution is applied to the transformed feature map $X^{\prime }$. The non-strided convolution avoids reducing the spatial resolution of the feature map, thereby preserving more detailed information [17]. The structure of SPDConv is illustrated in Figure 7.

**Figure 8:**
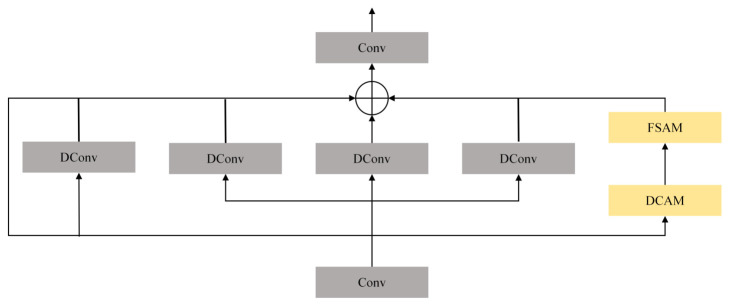
Structure diagram of the OmniKernel module.

**Figure 9:**
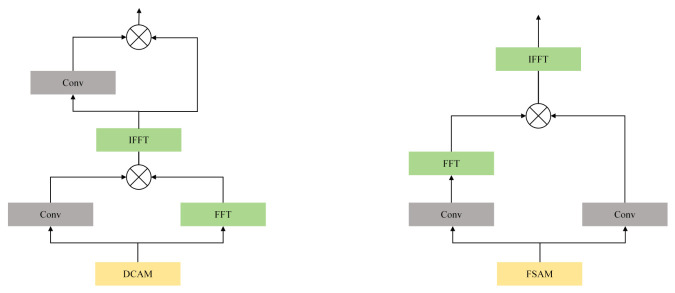
Structure: diagrams of DCAM and FSAM.

Next, the fused feature layer enters the Omni-Kernel module for further processing. The Omni-Kernel module consists of three main branches: a global branch, a large branch, and a local branch. The global branch captures extensive contextual information through a dual-domain channel attention mechanism (DCAM) and a frequency selective attention mechanism (FSAM), enabling the network to learn more comprehensive feature representations [18]. The large branch employs depthwise convolution and large kernels to provide multi-granularity receptive fields, further enriching the feature information; whereas the local branch concentrates on fine-grained local features through pointwise depthwise convolution, ensuring that small-scale information is fully preserved. The Omni-Kernel module and its related details are illustrated in Figure 8 and Figure 9.

**Figure 10:**
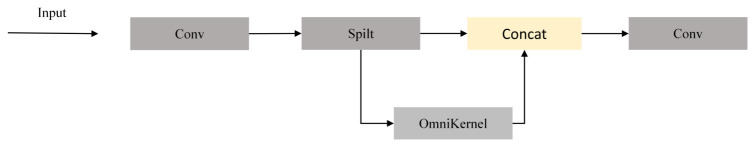
Structure diagram of the CSP-OmniKernel module.

The fused features are processed by the CSP-OmniKernel module. The CSP (Cross Stage Partial) structure splits the feature map into two parts: one part is passed through directly, while the other part undergoes a series of convolution and fusion operations before being integrated. This design makes feature fusion more efficient without significantly increasing the computational load [19]. The CSP-OmniKernel module is shown in Figure 10.

The fused feature map then undergoes an upsampling operation to elevate the low-resolution feature map to the same scale as the high-resolution feature map, thereby preserving more detailed information. The upsampling operation can be implemented through interpolation or deconvolution, restoring the scale of the feature map to its original size and further extracting the semantic information of small targets. Finally, the processed feature map is fused with the shallow features from the backbone network to refine the semantic and positional information of small targets. The fused feature map is subsequently processed by a decoupling head to output the final detection results. In this manner, SOEP can effectively enhance the detection performance of small targets without significantly increasing the computational burden.

### 2.5. Inner-MPDIoU

**Figure 11:**
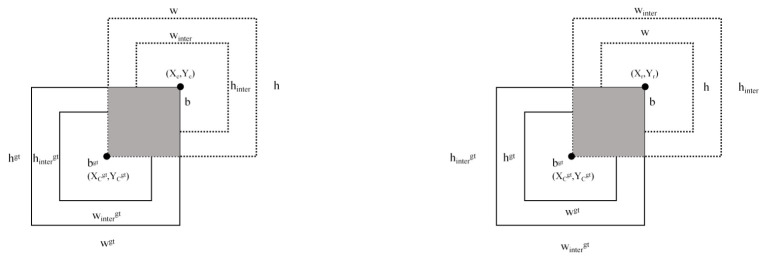
Schematic diagram of Inner-IoU.

Existing IoU-based bounding box regression methods primarily accelerate convergence by adding new loss terms, neglecting the inherent limitations of the IoU loss term itself; moreover, they fail to self-adjust across different detectors and detection tasks, resulting in weak generalization capability. By analyzing bounding box regression models, the Inner-IoU paper discovered that for high-IoU samples, using a smaller auxiliary bounding box to compute the loss can accelerate convergence, whereas a larger auxiliary bounding box is suitable for low-IoU samples. Therefore, referring to the Inner-IoU paper, this study computes the loss via an auxiliary bounding box, the size of which is primarily controlled by the ratio parameter [20], as illustrated in Figure 11.

The ground truth (GT) box and the anchor box are denoted as $B^{gt}$ and *B*, respectively; the center points of the GT box and its inner box are represented by $\left(x_{c}^{gt},y_{c}^{gt}\right)$, while $\left(x_{c},y_{c}\right)$ denotes the center points of the anchor box and its inner anchor box. The width and height of the GT box are represented as $w^{gt}$ and $h^{gt}$, respectively, and the width and height of the anchor box are represented as *w* and *h*, respectively. When the predicted box and the ground truth box share the same aspect ratio but differ in width and height values, the current loss function still presents issues; therefore, we further referred to the solution approach of MPDIoU, where in Equation (2), $\left(x_{1}^{A},y_{1}^{A}\right)$ and $\left(x_{2}^{A},y_{2}^{A}\right)$ represent the coordinates of the top-left and bottom-right corners of the yellow box, and $\left(x_{1}^{B},y_{1}^{B}\right)$ and $\left(x_{2}^{B},y_{2}^{B}\right)$ represent the coordinates of the top-left and bottom-right corners of the red box. $d_{1}$ and $d_{2}$ denote the squared distances between the two points, respectively. *w* and *h* refer to the width and height of the current feature map [21].

(2) $$\begin{array}{cc} d_{1} & ={\left(x_{1}^{B}-x_{1}^{A}\right)}^{2}+{\left(y_{1}^{B}-y_{1}^{A}\right)}^{2}\\ d_{2} & ={\left(x_{2}^{B}-x_{2}^{A}\right)}^{2}+{\left(y_{2}^{B}-y_{2}^{A}\right)}^{2} \end{array}$$

(3) $$\mathrm{Inner}\_\mathrm{MPDIoU}=\mathrm{inner}\_\mathrm{iou}-\frac{d_{1}}{w^{2}+h^{2}}-\frac{d_{2}}{w^{2}+h^{2}}$$

Finally, this study combines Inner-IoU with MPDIoU to propose the Inner-MPDIoU loss function. Targeting the current *Pleurotus ostreatus* quality grading dataset, this method employs different scale factors to control the generation of auxiliary bounding boxes of varying scales for experimentation, thereby overcoming the generalization limitations of existing methods and accelerating the bounding box regression process. Moreover, by directly computing the key point distances between the predicted box and the ground truth box, it more accurately reflects the disparity between them, simplifies the similarity comparison between two bounding boxes, and provides a more precise loss calculation result. The Inner-MPDIoU loss function integrates the scale-adaptive auxiliary bounding box strategy with the minimum point distance optimization method, thereby achieving faster convergence speed and more accurate regression results [22].

### 2.6. Random Pruning Method

To achieve real-time quality detection of *Pleurotus ostreatus* on resource-constrained edge devices, model lightweighting is an indispensable step to maintain optimal real-time performance [2]. Traditional pruning methods, such as $L_{1}$-Norm pruning or gradient-based sensitivity pruning, typically rely on complex “importance scoring” calculations [23]. However, the underlying assumption that channels with smaller absolute weight values or lower gradient contributions are less important does not always hold true in the non-convex optimization landscape of deep neural networks, and the scoring process itself introduces substantial computational overhead [12]. To circumvent these limitations, this study adopts a more concise and efficient Random channel pruning strategy to optimize the improved RTDETR model, minimizing computational complexity while maintaining high detection accuracy.

**Figure 12:**
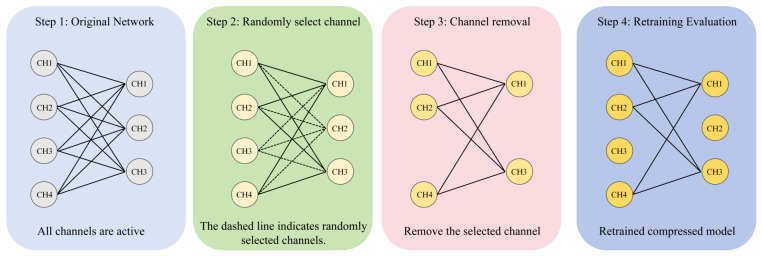
Explanatory diagram of the Random channel pruning principle.

Based on the “Lottery Ticket Hypothesis” and the over-parameterization characteristics of deep networks, the core premise of random pruning is that network performance primarily depends on its topological architecture and parameter space connectivity, rather than the explicit weight values of any single channel. For architectures like RTDETR that exhibit significant channel redundancy, randomly removing a certain proportion of channels essentially functions as a form of structural Dropout. This approach not only shrinks the computational footprint but also provides a regularizing effect that prevents overfitting. As illustrated in Figure 12, the implementation framework is structured into three main stages:

1. **Random Mask Generation**: Given a predefined pruning rate α, a binary mask vector $M_{l}\in {\left\{0,1\right\}}^{C_{l}}$ following a Bernoulli distribution is generated for each convolutional or linear layer *l*, where $C_{l}$ represents the total number of channels in that layer and the probability of pruning a channel satisfies $P(M_{li}=0)=\alpha$ [24].
2. **Structured Elimination**: The mask $M_{l}$ is applied to the corresponding weight matrix to physically eliminate channels with a mask value of 0 along with their associated input-output connections. Rather than merely masking weights to zero, this structured removal reconstructs a slimmed-down network layout, directly compressing the model’s parameters and FLOPs.
3. **Knowledge Fine-tuning**: Because random pruning alters original feature propagation pathways, it causes a temporary decline in model accuracy. To recover performance, the pruned network undergoes fine-tuning training on the training set using an SGD optimizer. This allows the remaining parameters to re-adapt and seek an optimal solution within the new topology, effectively restoring or even surpassing the baseline model’s capabilities.

Compared with conventional importance-based algorithms, random channel pruning offers low computational overhead, simple implementation, and exceptional robustness, while avoiding the risk of mistakenly deleting critical features due to erroneous importance estimations. Although omitting channel importance information historically carried the risk of severe feature loss, experimental results demonstrate that the fine-tuned randomly pruned model maintains extremely high average precision while significantly lowering the computational load, proving its efficacy and advancement in oyster mushroom edge detection scenarios.

## 3. Results

### 3.1. Experimental Environment

The experimental environment used in this study is shown in Table 3, and the testing environment is shown in Table 4.

### 3.2. Evaluation Metrics

Precision (*P*) refers to the proportion of samples correctly predicted as positive by the model among all samples predicted as positive, reflecting the classifier’s ability to avoid false positives [25]; the formula is:

(4) $$\mathrm{Precision}=\frac{\mathrm{TP}}{\mathrm{TP}+\mathrm{FP}}$$

Recall (*R*) refers to the proportion of actual positive samples that are correctly identified, reflecting the model’s sensitivity in detecting the target category; the formula is:

(5) $$\mathrm{Recall}=\frac{\mathrm{TP}}{\mathrm{TP}+\mathrm{FN}}$$

Mean Average Precision (mAP) is used to measure the average accuracy of the model across different categories. mAP is the mean of the average precision values across multiple categories [26,27]. The formulas are:

(6) $$\mathrm{AP}=\int_{0}^{1}P\left(R\right)\;dR$$

(7) $$\mathrm{mAP}=\frac{1}{|Q_{R}|}\sum_{q\in Q_{R}}\mathrm{AP}\left(q\right)$$

where $|Q_{R}|$ denotes the number of target categories, *q* denotes the category of the detection target, and AP(q) denotes the AP value for category *q*.

Parameters (Params): Params are used to evaluate the spatial complexity of the model [28].

Giga Floating-point Operations: GFLOPs is employed to assess the computational complexity of the model [29].

### 3.3. Comparison of Different Loss Functions

**Figure 13:**
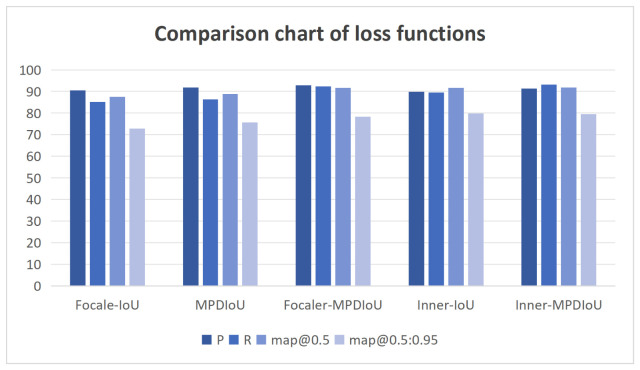
Comparison chart of loss functions.

To evaluate the impact of different loss functions on model performance, we conducted multiple experiments using the following loss functions: Focaler-IoU, MPDIoU, Focaler-MPDIoU, Inner-IoU, and Inner-MPDIoU. Table 5 and Figure 13 summarize the experimental results of each loss function.

First, we compared five different loss functions, including Focaler-IoU, MPDIoU, Focaler-MPDIoU, Inner-IoU, and Inner-MPDIoU. Each loss function possesses unique characteristics and application scenarios, influencing the speed of model training and detection accuracy. In this experiment, these loss functions were employed for training to evaluate their effectiveness in the detection of *Pleurotus ostreatus* fruiting bodies.

The Focaler-IoU loss function performed excellently in balancing the weights of hard and easy samples, but showed mediocre performance in improving the precision of high-IoU samples. The MPDIoU loss function, on the other hand, could better capture the details between the predicted box and the ground truth box when handling bounding box regression, significantly improving the model’s precision and recall rate. The effectiveness of MPDIoU was particularly pronounced for targets with complex morphological characteristics. The Focaler-MPDIoU method, which combines Focaler and MPDIoU, further improved the model’s performance in complex detection tasks by enhancing the weight balance of hard and easy samples while simultaneously optimizing bounding box regression.

In contrast, the Inner-IoU and Inner-MPDIoU methods exhibited better convergence; in particular, the Inner-MPDIoU loss function further optimized the object detection process by finely adjusting the relative position between the predicted box and the ground truth box. Although Focaler-MPDIoU achieved the highest precision (92.8%) and Inner-IoU slightly led in the stringent mAP@0.5:0.95 metric (79.9% vs. 79.5%), Inner-MPDIoU was definitively selected as the optimal loss function due to its superior comprehensive performance. When determining the final loss function, the primary consideration was the overall balance of detection metrics, particularly the trade-off between Precision (P) and Recall (R). In the practical morphological evaluation of *Pleurotus ostreatus*, minimizing missed detections (yielding a high Recall) is crucial for subsequent commercial grading. Compared to Inner-IoU, Inner-MPDIoU brings a substantial 3.6% improvement in Recall (from 89.5% to 93.1%) and a 1.5% increase in Precision (from 89.9% to 91.4%), alongside a slight gain in mAP@0.5 (91.9% vs. 91.7%). Therefore, the negligible 0.4% trade-off in mAP@0.5:0.95 is entirely justified by the significant enhancements in precision, recall, and overall detection robustness, making Inner-MPDIoU the most balanced and practical bounding box regression strategy for the morphological quality detection of *Pleurotus ostreatus*.

### 3.4. Pruning Experiments

The purpose of the pruning experiments is to evaluate the optimization of the model’s computational efficiency by reducing the computational load and parameter count while maintaining model accuracy. In this study, we adopted the Random channel pruning method, which reduces redundant computation by randomly removing a portion of channels in the neural network while preserving the core features of the model.

By comparing the effects of different pruning strategies, the Random pruning method demonstrated significant advantages in improving computational efficiency. Although the model’s parameter count was reduced, its performance in terms of precision and recall was hardly affected, and in some cases, precision and recall even improved. This indicates that pruning can not only effectively reduce the model’s computational complexity but can also enhance model performance in certain cases; particularly in resource-constrained environments, pruning technology provides significant assistance in improving real-time performance.

**Figure 14:**
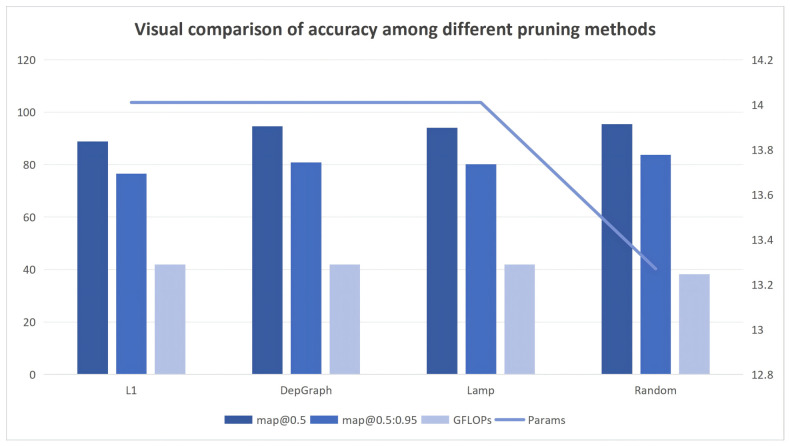
Visual comparison of accuracy among different pruning methods.

Compared with other pruning methods, although L1 pruning and DepGraph pruning can also reduce the computational load, they suffer from a certain loss in accuracy. In contrast, Random pruning achieved a better balance between parameter count and GFLOPs, demonstrating lower computational overhead and higher detection accuracy. Especially for relatively small and complex targets such as *Pleurotus ostreatus* fruiting bodies, the Random pruning method was able to significantly improve the model’s inference speed without sacrificing performance; it performed most outstandingly among multiple pruning strategies, achieving the lowest GFLOPs (38.2) and the highest precision and recall in extreme pruning. This demonstrates that pruning can significantly enhance the model’s computational efficiency while maintaining high detection accuracy.For details, please refer to Table 6 and Figure 14.

### 3.5. Ablation Experiments

In the ablation experiments, we progressively removed different components of the model to analyze the contribution of each component to the overall model performance. These experiments included modules such as FasterNet, SOEP, Inner-MPDIoU, and the pruning method; by removing these components one by one, their impact on model accuracy and efficiency was evaluated. As a core part of the model, FasterNet is responsible for feature extraction and network structure optimization, significantly improving the training speed and inference efficiency of the network. However, when FasterNet was used alone, although the model accuracy reached a certain level, its performance was slightly inadequate when facing complex scenes and small targets. Next, we introduced the SOEP (Small Object Enhancement Pyramid) module, which performed particularly prominently when handling small targets, effectively capturing the detailed features of small targets and improving detection accuracy through feature fusion. After combining with FasterNet, the overall performance of the model was improved, especially in terms of mAP@0.5:0.95.

**Figure 15:**
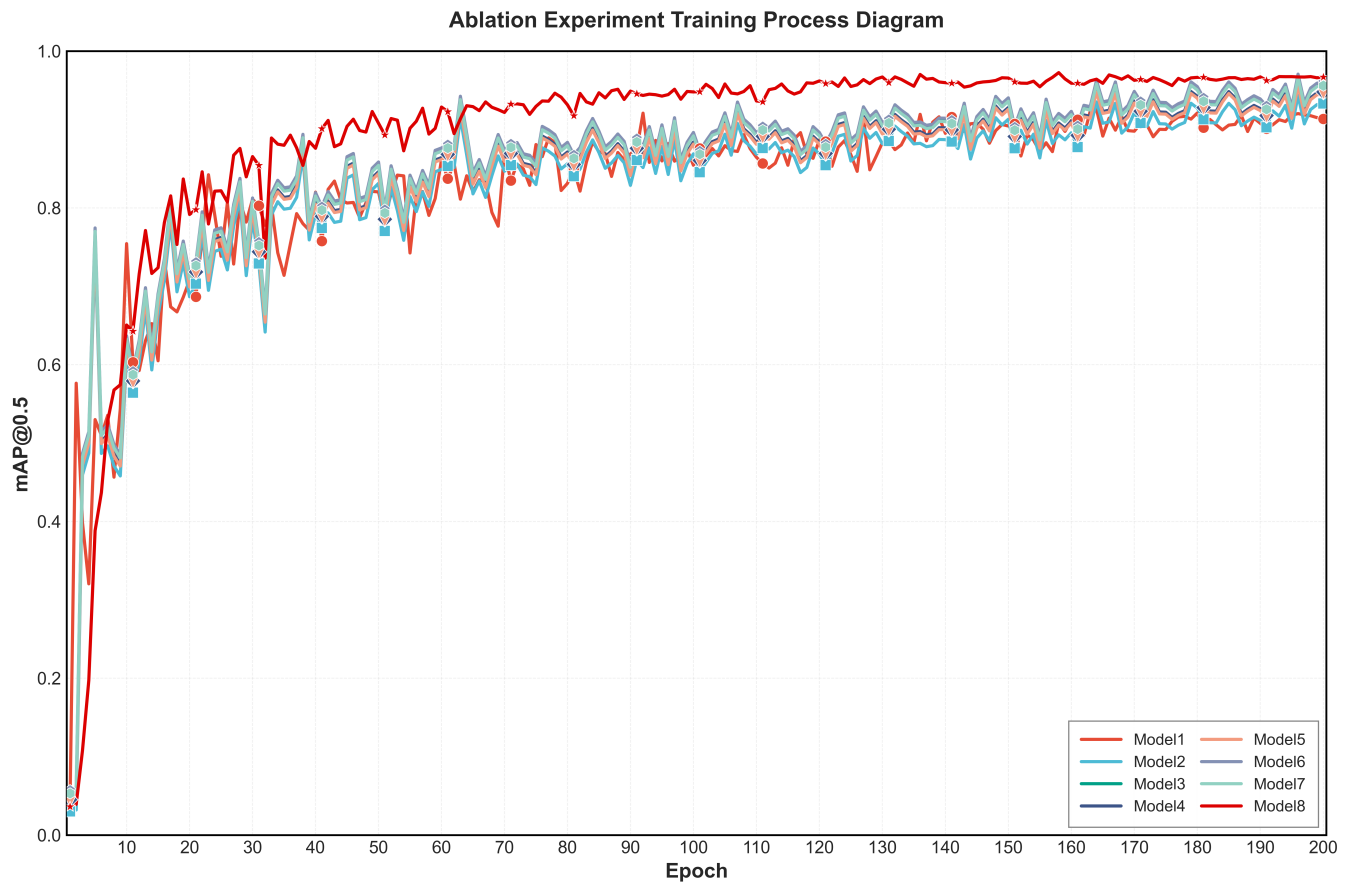
Training plots of each process in the ablation experiments.

The Inner-MPDIoU module further enhanced the model’s accuracy in bounding box regression, optimizing the target localization precision. After integrating this module with FasterNet and SOEP, the model demonstrated significant improvements in both detection accuracy and recall rate compared to the baseline models. Removing any single module led to a decline in model performance; particularly, after removing the SOEP module, the model’s detection capability for small targets was significantly weakened. Therefore, the results of the ablation experiments indicate that the combination of FasterNet, SOEP, and Inner-MPDIoU is the key factor in improving the detection accuracy and efficiency of *Pleurotus ostreatus* fruiting bodies.For full details, see Table 7 and Figure 15.

### 3.6. Comparative Experiments

To verify the efficacy of POC-DETR-Prune in detecting the morphological quality of *Pleurotus ostreatus* fruiting bodies, horizontal comparative experiments were systematically conducted against mainstream object detectors. The baseline models included convolutional neural networks from the YOLO series (YOLOv5m, YOLOv8m, YOLO11m, and YOLO12m) and the original RTDETR series (RTDETR-l, RTDETR-R34, and RTDETR-R50). For fairness, all models were evaluated under identical hardware and software environments using a multi-dimensional suite of metrics: Precision (P), Recall (R), mAP@0.5, mAP@0.5:0.95, Parameters (Params), and GFLOPs.

Quantitative results summarized in Table 8 demonstrate that POC-DETR-Prune delivers superior detection accuracy, achieving 95.5% mAP@0.5 and 83.7% mAP@0.5:0.95. Compared to YOLO12m, our model improves mAP@0.5 by 1.8 percentage points. This performance leap stems from the synergistic integration of the Inner-MPDIoU loss and the SOEP module, which mitigate localization drift caused by overlapping caps and preserve fine-grained texture features. Crucially for harvesting automation, POC-DETR-Prune achieves a Recall rate of 96.7%, significantly reducing missed detections in dense, mutually occluded mushroom clusters compared to YOLOv8m (84.3%) and RTDETR-l (90.7%).

The architectural divergence between our model and the CNN-based YOLO series underscores a clear performance watershed. While the optimized CSP and ELAN modules in recent YOLO iterations improve feature aggregation, CNNs remain constrained by local receptive fields. Consequently, they struggle with classification confusion in complex backgrounds when distinguishing highly similar morphological grades (e.g., YOLOv5m only achieved 76.7% on mAP@0.5:0.95). In contrast, POC-DETR-Prune exploits the global attention mechanism of the Transformer architecture alongside FasterNet to capture long-range dependencies across the image, enabling precise differentiation of subtle phenotypic variations.

**Figure 16:**
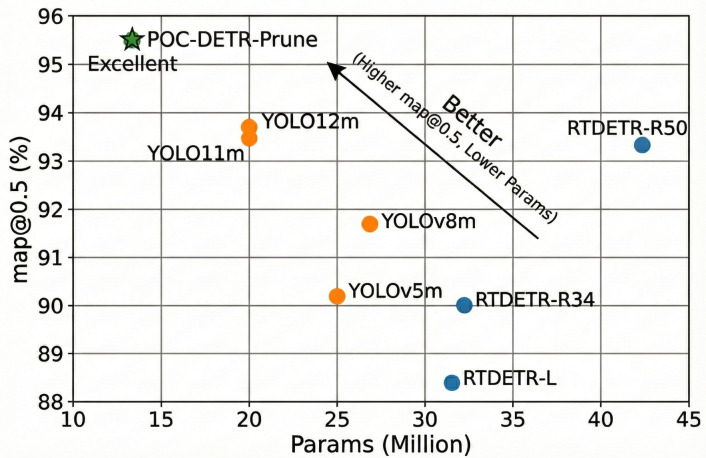
Scatter plot of the relationship between mAP@0.5 and parameter count for each model.

**Figure 17:**
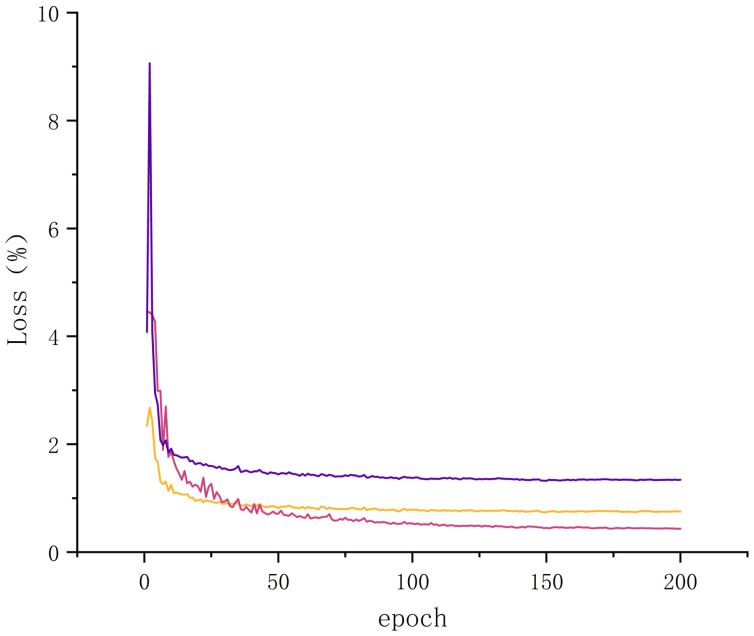
Training loss plot of POC-DETR-Prune.

The performance-parameter trade-off is visually summarized in Figure 16, where POC-DETR-Prune occupies the ’excellent’ region, achieving the highest mAP@0.5 with the fewest parameters compared to the YOLO and original RT-DETR series models. This clear separation in the scatter plot demonstrates the superior efficiency of our proposed optimization strategy. Furthermore, Figure 17 illustrates the training loss convergence curves for POC-DETR-Prune. The plot exhibits a stable and rapid decline in training loss, reaching a stable state within 200 epochs, which confirms the effectiveness and training stability of the collaborative optimization modules introduced in our framework.

**Figure 18:**
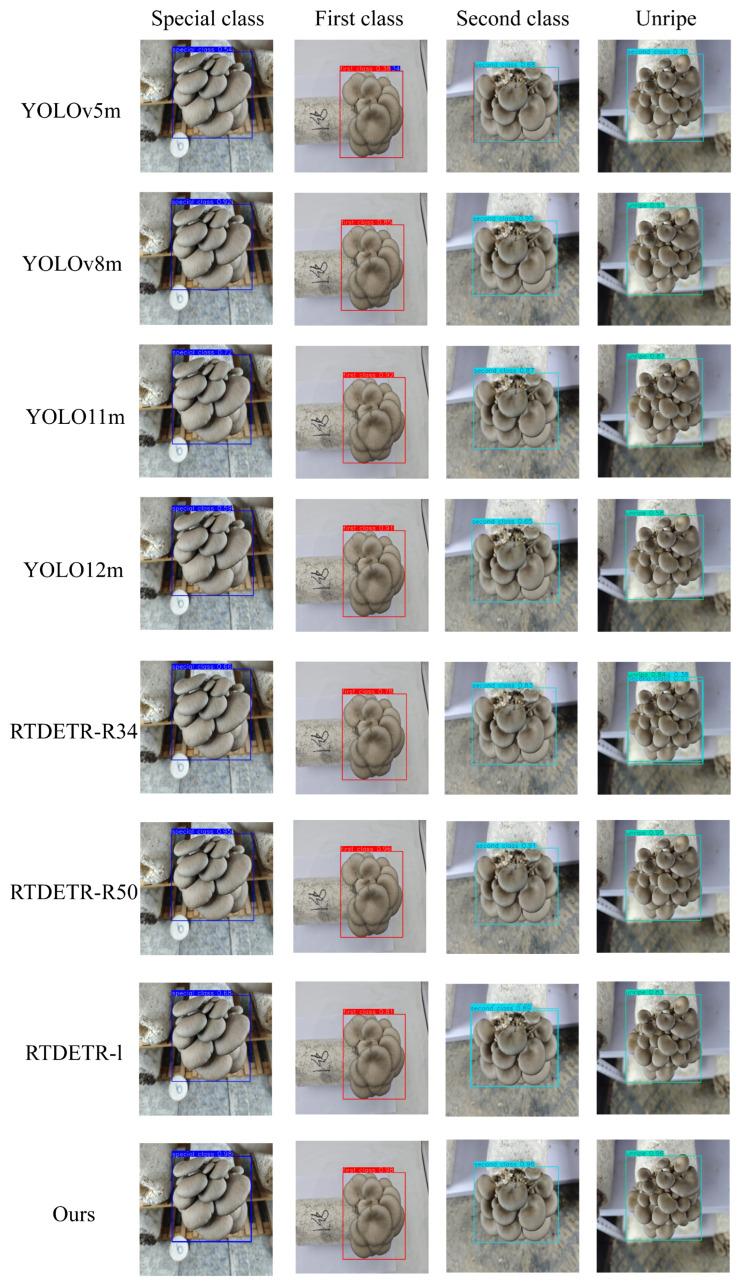
Comparison chart of detection results for each model.

Comparisons with the original RTDETR series validate that model lightweighting does not necessitate performance degradation. The baseline RTDETR-R50 delivers 81.3% mAP@0.5:0.95 but demands 41.96 M parameters and 129.6 GFLOPs. Through Random channel pruning and structural restructuring, POC-DETR-Prune compresses the parameter count to 13.27 M (31.6% of R50) and the computational load to 38.2 GFLOPs (29.5% of R50), while outperforming R50 in accuracy by 2.4 percentage points. Qualitative visualizations corroborate these quantitative findings. As illustrated in Figure 18, conventional models exhibit missed detections under uneven illumination and dense clustering. Conversely, POC-DETR-Prune consistently achieves robust localization and highly reliable bounding box regression across all complex operational scenarios.

### 3.7. Detailed Class-Wise Performance and Confusion Matrix Analysis

To clarify the specific composition of the quality grading framework and illustrate how grading decisions are derived for the entire fruiting body cluster, a detailed class-wise performance evaluation was conducted. Table 9 summarizes the Precision, Recall, Average Precision at an IoU threshold of 0.5 (AP@50), and the AP averaged over multiple IoU thresholds (AP@50:95) for each of the four designated classes (Special Class, First Class, Second Class, and Unripe).

As presented in the table, the overall mAP@50 of the model reaches 95.5%, and the mAP@50:95 reaches 83.7%. The model’s performance on the AP@50:95 metric verifies the effectiveness of the Inner-MPDIoU loss function in improving bounding box regression accuracy, which meets the requirement for quantifying cap dimensions in densely clustered mushrooms. Among them, the Second Class and Unripe categories achieved AP@50:95 scores of 84.6% and 84.3%, respectively.

**Figure 19:**
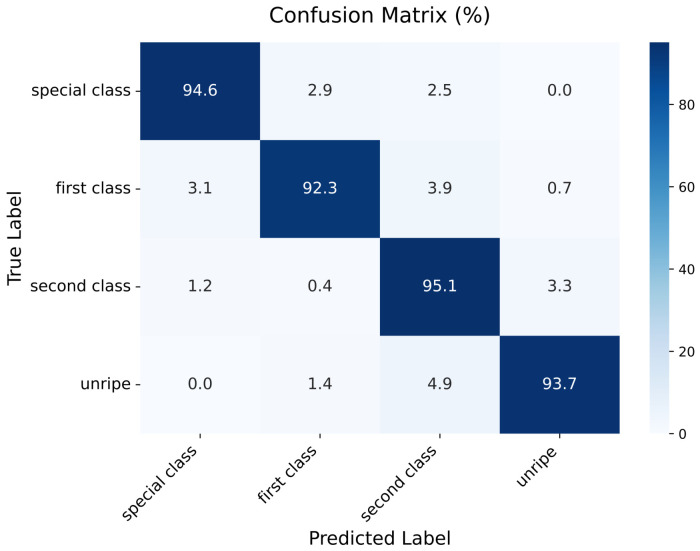
Confusion matrix of the proposed POC-DETR-Prune model for *Pleurotus ostreatus* quality grading.

To further analyze the classification boundaries and error distributions of the proposed model across different commercial production units, the corresponding normalized confusion matrix is shown in Figure 19.

The diagonal elements of the confusion matrix illustrate the classification accuracy of the model across all categories. Specifically, the correct identification rates for the Special Class, First Class, Second Class, and Unripe categories are 94.6%, 92.3%, 95.1%, and 93.7%, respectively. The primary source of classification error is concentrated between adjacent quality grades. For example, 2.9% of Special Class clusters are misclassified as First Class, while 3.9% of First Class clusters are misclassified as Second Class.

This misclassification phenomenon is reasonable in biology and practical applications, as the transitional morphological characteristics between adjacent classes represent a continuous gradual process rather than discrete boundaries. Due to its characteristic tightly clustered morphology and smaller cap dimensions, the Unripe class maintains a higher recall (96.1%) and exhibits relatively less confusion with mature classes. In summary, the diagonal accuracy of the confusion matrix and its error distribution indicate that the proposed model can provide a reliable decision reference for automated edge-sorting equipment in daily production and life.

### 3.8. Generalization Validation

To verify the stability and robustness of the model, we first employed the *k*-fold cross-validation method to evaluate the model’s performance under different data splits. The experimental results indicate that the model performed stably across different folds. This demonstrates that our model possesses strong generalization capability across multiple datasets and can adapt to detection tasks under different environments and conditions. Through cross-validation, we can effectively reduce the model’s dependence on a single dataset, ensuring its reliability and stability in practical applications. Especially in agricultural production environments, the diversity and complexity of data require the model to adapt to different cultivation conditions and external factors, and it is precisely in this regard that POC-DETR-Prune exhibits strong adaptability.See the specific results Table 10.

**Figure 20:**
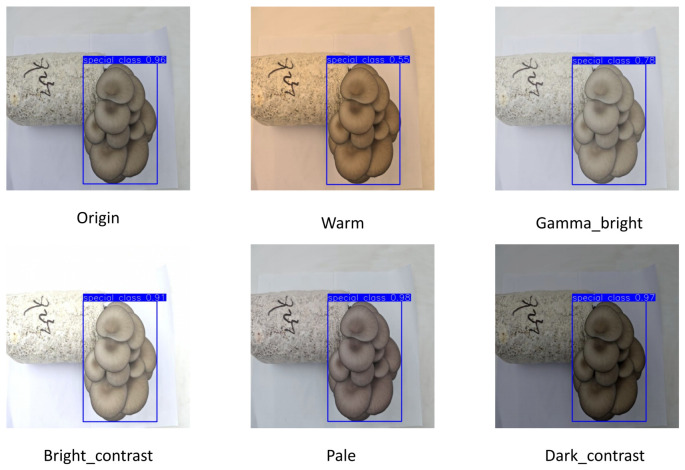
Detection performance under various illumination and color tone augmentations.

Meanwhile, to further validate the generalization capability of the POC-DETR-Prune model, this study randomly selected images from the test set and applied several data augmentations, primarily including Warm, Pale, Bright, and Dark transformations. The detection results under these conditions are shown in Figure 20. These augmentations simulate significant environmental shifts that may occur in practical production. Although the induced visual perturbations naturally affect the model’s prediction confidence compared to ideal imaging conditions, all detected targets in Figure 20 consistently maintain confidence scores above the predefined threshold of 0.25. The model’s ability to successfully retain and correctly classify dense clusters under these visual variations proves its practical adaptability and generalization capability in complex environments.

**Figure 21:**
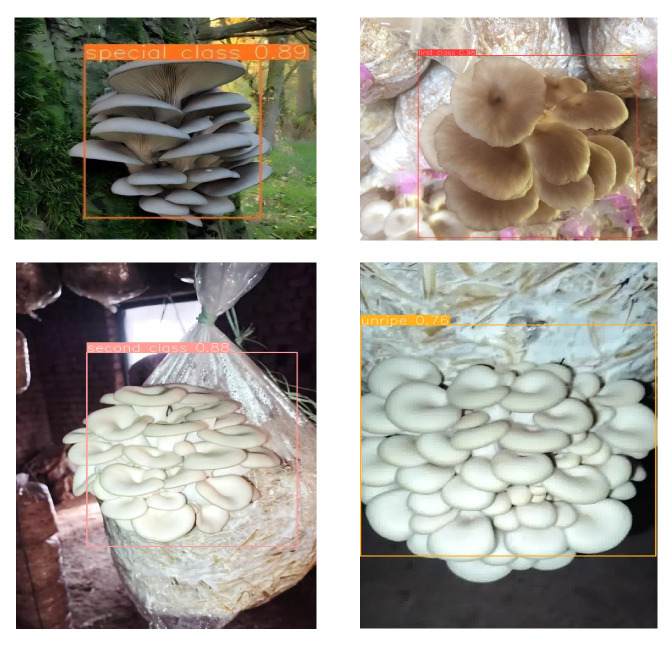
External test set results image.

To explicitly address the requirement for the model to adapt to various production environments when achieving factory-scale deployment, this study constructed and evaluated an external test set. The images in this external dataset were all collected from environments completely different from those in the model training and testing phases. As shown in Figure 21, compared to the original dataset, these images feature diverse background interferences, cultivation layouts, focal distances, and ambient lighting conditions. Despite these severe cross-environment differences, the POC-DETR-Prune model can still provide highly accurate bounding box localization and reliable morphological grading for the *Pleurotus ostreatus* clusters. The robust performance on the external test set proves that the proposed model does not overfit to specific data sources, but rather possesses a certain degree of practical environmental adaptability required for commercial factory-scale edge deployment.

### 3.9. Edge Device Deployment and Performance Comparison

To evaluate the practical applicability of the proposed model in real-world agricultural environments, comprehensive deployment tests were conducted on the NVIDIA Jetson Orin Nano edge computing platform. To ensure fairness and maximize hardware efficiency, all models were optimized using the TensorRT inference accelerator [30]. The uniform testing configurations were strictly defined as follows: the input resolution was set to 640×640, the batch size was 8, and the precision mode was configured to half-precision floating-point (FP16). Furthermore, the power consumption mode of the edge device was locked at 25 W throughout the tests.

It is important to note that the benchmark metrics were recorded under pure inference conditions (without the I/O overhead of calling a physical camera) to accurately reflect the algorithmic efficiency. The deployment performance comparison of various object detection models is summarized in Table 11.

As illustrated in Table 11, the proposed POC-DETR-Prune model exhibits substantial advantages across all edge deployment metrics. Its pure inference speed reaches 46.94 FPS with a latency of only 21.8 ms, and its memory footprint is effectively constrained to 3.24 GB. Compared to the original RTDETR series, our model more than doubles the processing speed while reducing memory consumption by over 60%. Even when compared to the highly optimized YOLO series (such as YOLO11m and YOLO12m), POC-DETR-Prune consistently maintains noticeable superiority in speed, latency, and memory conservation.

**Figure 22:**
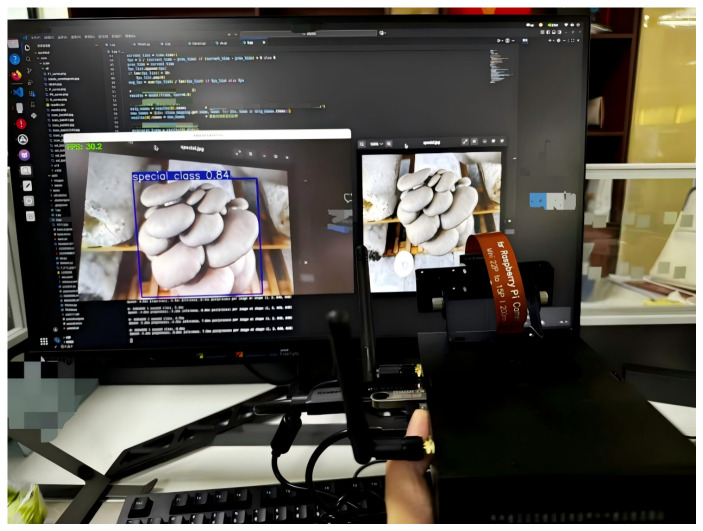
Actual deployment diagram of POC-DETR-Prune.

Moreover, in practical production tests involving continuous video streaming from industrial cameras—which naturally introduces additional I/O overhead for image acquisition and preprocessing—the POC-DETR-Prune model reliably sustains an operational speed of approximately 30 FPS. This performance fully satisfies the stringent real-time requirements for the commercial morphological quality grading of *Pleurotus ostreatus*. Actual deployment diagram of POC-DETR-Prune (see Figure 22).

## 4. Discussion

This study constructed a multi-module collaboratively optimized POC-DETR-Prune model based on the RTDETR framework to meet the requirements for intelligent detection of the morphological quality of *Pleurotus ostreatus* fruiting bodies. Through strategic advancements in feature extraction, small target perception, bounding box regression, and model lightweighting, the proposed framework exhibits robust and competitive performance in complex operational environments.

### 4.1. Analysis of the Impact of Core Modules on Detection Performance

For feature extraction, the FasterNet module utilizes Partial Convolution (PConv) to optimize memory access, enhancing operator scheduling efficiency during large-scale computation. Results show that FasterNet provides clearer feature representations for Transformer decoding, ensuring stability in texture-dense regions like cap edges and stipe details. To address small target occlusion and feature loss from clustered growth, the SOEP module incorporates high-resolution P2 layer features with SPDConv mAPping, preserving fine-grained details in deep networks. Ablation experiments reveal that integrating the SOEP module alone improves mAP@0.5:0.95 by 0.8%, demonstrating that its parallel global, large-kernel, and local branches are irreplaceable for capturing complex morphological semantics. Regarding localization, the Inner-MPDIoU loss leverages point distance constraints and an auxiliary bounding box mechanism to overcome the gradient vanishing problem inherent in traditional IoU loss at high IoU stages, outperforming MPDIoU and Focaler-MPDIoU in irregular edge and boundary blurring scenarios.

### 4.2. Comparative Analysis with Existing Research

Horizontal comparisons with related works highlight the practical value of this study. Unlike Qi et al. [3], who analyzed growth states via Mamba-YOLO, or Shi et al. [6], whose OMC-YOLO model focused exclusively on individual mushroom pieces, this study targets the comprehensive grading of *Pleurotus ostreatus* based on national standards. In terms of accuracy, while Xie et al. [5] achieved a mAP50:95 of 0.743 for enoki mushroom grasping segmentation, our model reaches a mAP@0.5:0.95 of 83.7% in dense, highly overlapped scenarios, showing the clear advantage of our improved bounding box regression mechanism. For lightweighting, although Shi et al. [4] reduced parameters by modifying YOLOv8n, our approach incorporates FasterNet and Random channel pruning within the RTDETR architecture to optimize the computational load from 129.6 GFLOPs to 38.2 GFLOPs. The fine-tuned, pruned model achieves a computational cost of only 66.8% of the original architecture while maintaining accuracy, making it highly suitable for embedded devices like farm grading machines and intelligent harvesting robots.

### 4.3. Generalization Capability and Application Prospects

Multiple comparative experiments confirm that POC-DETR-Prune outperforms the YOLOv5, YOLOv8, and YOLO11 series. The *k*-fold cross-validation stable performance underscores its dataset independence and strong generalization across diverse environmental conditions. This multi-module collaborative framework balances speed, precision, and efficiency, offering scalability to other complex agricultural vision tasks. In practical engineering, its low computational requirement (38.2 GFLOPs) allows seamless deployment on resource-constrained platforms like the NVIDIA Jetson Orin Nano Super, decoupling terminal device operations from high-performance servers. The model has been verified to achieve over 20 FPS on embedded systems, providing robust underlying algorithmic support for smart agricultural edge automation equipment.

## 5. Conclusions

This study addresses the critical need for automated, non-destructive quality evaluation in the food industry by proposing the POC-DETR-Prune model for *Pleurotus ostreatus* grading. By integrating the FasterNet feature extraction network, the SOEP module, and the Inner-MPDIoU loss function, the model overcomes the challenges of accurately profiling clustered and highly overlapped food matrices. The proposed methodology achieved a high accuracy of 83.7% (mAP@0.5:0.95) and a recall rate of 96.7%, outperforming mainstream models such as YOLO12m. Crucially for industrial application, a Random channel pruning strategy reduced the computational load to 38.2 GFLOPs. Deployed on an NVIDIA Jetson Orin Nano Super edge computing platform, the system realized a real-time processing speed of 30.2 FPS. This emerging technological solution successfully balances high-precision morphological grading with the lightweight hardware requirements of modern factories, paving the way for advanced, real-time automated sorting equipment in the edible fungi processing sector.

## Figures and Tables

**Table 1:** Grading standards for *Pleurotus ostreatus*.

Standard	Special Class	First Class	Second Class
**Color **	Possesses the natural color of the variety, with uniform and consistent luster, a smooth and clean cap surface, and no heterochromatic spots.	Possesses the natural color of the variety, with relatively uniform and consistent luster, a smooth cap surface, and slight heterochromatic spots are permitted.	Possesses the natural color of the variety, with basically uniform and consistent luster, a relatively smooth and clean cap surface, with slight heterochromatic spots.
**Morphology **	Fan-shaped or palm-shaped, with an involute cap margin, thick flesh, a cleanly cut stipe base, and no water-soaked appearance or slimy feel.	Fan-shaped or palm-shaped, with a slightly expanded cap margin, thick flesh, a relatively cleanly cut stipe base, and no water-soaked appearance or slimy feel.	Fan-shaped or palm-shaped, with a slightly expanded cap margin, and slight irregularities are permitted at the stipe base.

**Table 2:** Distribution of dataset classes before and after augmentation.

Class	Original Dataset	Augmented Dataset
Special Class	397	1291
First Class	538	1276
Second Class	610	1220
Unripe	366	1253
Total	1911	5040

**Table 3:** Overview of the experimental environment.

Component	Specification
CPU	Intel(R) Xeon(R) Gold 5218 CPU @ 2.30 GHz
GPU	NVIDIA GeForce RTX 4060 Ti 16 GB × 2
Memory	64 GB (Training)
Operating System	Windows 10
Framework	PyTorch 2.1.2
Programming Language	Python 3.8
CUDA Version	11.8
Random seed	42

**Table 4:** Overview of the testing environment.

Component	Specification
Main Configuration	NVIDIA Jetson Orin Nano Super Development Kit, (NVIDIA, Santa Clara, CA, USA)
Memory	8 GB
Operating System	Ubuntu 22.04
TensorRT Version	TensorRT 12
Framework	PyTorch 2.1.2
Programming Language	Python 3.10
CUDA Version	11.8
Camera	CLB IMX219

**Table 5:** Performance of different loss functions.

Loss Functions	P (%)	R (%)	mAP@0.5 (%)	mAP@0.5:0.95 (%)
Focaler-IoU	90.5	85.1	87.5	72.9
MPDIoU	91.9	86.3	88.9	75.7
Focaler-MPDIoU	92.8	92.3	91.6	78.3
Inner-IoU	89.9	89.5	91.7	79.9
Inner-MPDIoU	91.4	93.1	91.9	79.5

**Table 6:** Performance of different pruning methods.

Method	P (%)	R (%)	mAP@0.5 (%)	mAP@0.5:0.95 (%)	Params (M)	GFLOPs	FPS	Latency (ms)
L1	92.6	87.0	88.9	76.6	14.01	41.9	55.3	16.2
DepGraph	91.9	96.1	94.6	80.8	14.01	41.9	57.6	16.7
Lamp	92.2	94.8	94.0	80.2	14.01	41.9	52.6	16.4
Random	93.9	96.7	95.5	83.7	13.28	38.2	53.7	16.5

**Table 7:** Summary of ablation experiments.

FasterNet	SOEP	Inner-MPDIoU	Prune	P (%)	R (%)	mAP@0.5 (%)	mAP@0.5:0.95 (%)	Params	GFLOPs
✓	✓	✓	✓	93.9	96.7	95.5	83.7	13,275,312	38.2
✓	✓	✓	×	92.9	94.6	95.0	82.9	17,404,832	57.7
✓	×	✓	×	90.9	93.1	92.4	79.6	16,788,896	49.5
×	✓	✓	×	92.8	96.0	94.8	82.4	20,492,832	65.2
✓	✓	×	×	93.2	94.7	94.3	81.3	17,404,832	57.7
×	×	✓	×	91.4	93.1	91.9	79.5	19,876,896	57.0
×	✓	×	×	91.0	92.1	91.4	78.4	20,492,832	65.2
✓	×	×	×	92.8	94.0	92.1	79.2	16,788,896	49.5
×	×	×	×	90.5	92.1	90.8	77.6	19,876,896	57.0

Where ✓ indicates that the component was used, and × indicates that the component was not used.

**Table 8:** Summary of parameters for each model.

Models	P (%)	R (%)	mAP@0.5 (%)	mAP@0.5:0.95 (%)	Params	GFLOPs
POC-DETR-Prune	93.9	96.7	95.5	83.7	13,275,312	38.2
RTDETR-l	89.4	90.7	88.5	76.1	31,992,216	103.4
RTDETR-R50	92.7	92.3	93.4	81.3	41,962,328	129.6
RTDETR-R34	89.4	91.2	90.0	78.9	31,110,856	88.8
YOLO12m	82.0	88.3	93.7	80.7	20,107,996	67.1
YOLO11m	87.5	85.1	93.5	79.8	20,033,116	67.7
YOLOv8m	86.0	84.3	91.6	76.9	25,842,076	78.7
YOLOv5m	84.7	85.5	90.2	76.7	25,047,532	64.0

**Table 9:** Detailed class-wise performance of the POC-DETR-Prune model.

Class	Precision (%)	Recall (%)	AP@50 (%)	AP@50:95 (%)
Special Class	94.6	97.2	94.8	82.8
First Class	92.3	96.5	96.5	82.9
Second Class	95.1	97.1	95.6	84.6
Unripe	93.7	96.1	95.2	84.3
**Average**	**93.9**	**96.7**	**95.5**	**83.7**

**Table 10:** *k*-fold cross-validation results.

Models	P (%)	R (%)	mAP@0.5 (%)	mAP@0.5:0.95 (%)
k-fold-1	93.9	96.7	95.5	83.7
k-fold-2	92.7	94.3	95.1	83.1
k-fold-3	91.3	94.1	94.5	83.3
k-fold-4	92.1	93.8	94.7	83.1
k-fold-5	92.7	93.8	95.1	83.3

**Table 11:** Performance comparison of different models deployed on the NVIDIA Jetson Orin Nano edge device (TensorRT optimized, FP16, Batch Size = 8, Resolution = 640×640).

Model	Power (W)	Memory Usage (GB)	Latency (ms)	FPS
RTDETR-l	25	6.85	30.4	16.65
RTDETR-R50	25	6.74	36.7	17.88
RTDETR-R34	25	5.69	33.5	20.50
YOLOv5m	25	3.73	26.9	39.73
YOLOv8m	25	4.11	28.6	38.29
YOLO11m	25	3.68	24.6	40.82
YOLO12m	25	3.92	26.7	39.66
**POC-DETR-Prune **	**25 **	**3.24 **	**21.8 **	**46.94 **

## Data Availability

The original contributions presented in this study are included in the article. Further inquiries can be directed to the corresponding authors.
